# Evaluation of a Chicken 600K SNP genotyping array in non-model species of grouse

**DOI:** 10.1038/s41598-019-42885-5

**Published:** 2019-04-23

**Authors:** Piotr Minias, Peter O. Dunn, Linda A. Whittingham, Jeff A. Johnson, Sara J. Oyler-McCance

**Affiliations:** 1 Department of Biodiversity Studies and Bioeducation, Faculty of Biology and Environmental Protection, University of Łódź, Banacha 1/3, 90-237 Łódź, Poland; 2 Behavioral and Molecular Ecology Group, Department of Biological Sciences, University of Wisconsin-Milwaukee, Milwaukee, Wisconsin USA; 3 Department of Biological Sciences, Institute of Applied Sciences, University of North Texas, Denton, Texas USA; 4 U.S. Geological Survey, Fort Collins Science Center, Ft. Collins, Colorado, USA

**Keywords:** Conservation genomics, Phylogenomics

## Abstract

The use of single nucleotide polymorphism (SNP) arrays to generate large SNP datasets for comparison purposes have recently become an attractive alternative to other genotyping methods. Although most SNP arrays were originally developed for domestic organisms, they can be effectively applied to wild relatives to obtain large panels of SNPs. In this study, we tested the cross-species application of the Affymetrix 600K Chicken SNP array in five species of North American prairie grouse (*Centrocercus* and *Tympanuchus* genera). Two individuals were genotyped per species for a total of ten samples. A high proportion (91%) of the total 580 961 SNPs were genotyped in at least one individual (73–76% SNPs genotyped per species). Principal component analysis with autosomal SNPs separated the two genera, but failed to clearly distinguish species within genera. Gene ontology analysis identified a set of genes related to morphogenesis and development (including genes involved in feather development), which may be primarily responsible for large phenotypic differences between *Centrocercus* and *Tympanuchus* grouse. Our study provided evidence for successful cross-species application of the chicken SNP array in grouse which diverged ca. 37 mya from the chicken lineage. As far as we are aware, this is the first reported application of a SNP array in non-passerine birds, and it demonstrates the feasibility of using commercial SNP arrays in research on non-model bird species.

## Introduction

DNA genotyping arrays with hundreds of thousands (or more) single nucleotide polymorphisms (SNPs) are commonly used in genetic studies of model species, but relatively few arrays have been developed in other species because of their high costs of development. In non-model species, alternative techniques such as Restriction Associated DNA sequencing (RAD-seq) are more often used to analyze SNPs, but for a variety of reasons studies using RAD-seq often have an order of magnitude fewer SNPs (tens of thousands of SNPs) than SNP arrays, which can be insufficient for some applications (e.g. genome-wide association studies, GWAS^1^). Studies using RAD-seq compared to SNP arrays also involve more extensive processing requiring specialized expertise to filter and identify genetic markers. Thus, some researchers have recently tested commercial SNP arrays developed for model species, particularly mammals, on closely related non-model species to determine if they can provide easier access to larger numbers of SNPs^2–4^. In contrast to mammals and plants, relatively few studies have applied SNP arrays to studies of birds. Some studies of passerine birds have used custom Illumina beadchip arrays^5–7^, but they are not commercially available and have relatively fewer SNPs (10–50 K) than arrays for other model organisms. Also, the passerine arrays may not be readily applicable to non-passerine birds, as the two lineages are estimated to have split 60–75 million years ago (mya)^8,9^. The most recent SNP array manufactured by Affymetrix for a non-passerine poultry species, the domestic chicken *Gallus gallus*, however, has nearly 600 000 SNPs including a set of SNPs in exonic or coding regions^10^ and could be potentially useful for applications to non-passerine birds.

One limitation of using SNP arrays is the level of cross-species amplification. In a study of 16 wild mammal species, the percentage of called SNPs declined 1.5% for each million years of evolutionary divergence^11^. Despite the apparent reduction in call rate, some SNP arrays have been successfully applied to phylogenetically distant lineages. For example, the EquineSNP50, developed for the domestic horse, still yielded a call rate of 25% when applied to species in the suborder Ceratomorpha (tapirs and rhinoceros), which diverged ca. 54 mya from the equine lineage^12^. On the other hand, cross-species application of the domestic dog CanineHD BeadChip to seals (divergence time ~44 mya) amplified 19% of 173 K loci, but fewer than 200 loci were polymorphic^13^. Another issue with cross-species use of SNP arrays (and other markers) is ascertainment bias, in which the sample of SNPs does not represent the overall pattern of genetic diversity, in this case because the SNP array was developed in a different species. This difference is likely to lead to an over-representation of polymorphisms shared between species or polymorphisms resulting from artificial selection, when SNPs originally developed for domestic species are cross-amplified in wildlife. Ascertainment bias might also lead to spatial clustering of polymorphisms around selectively non-neutral SNPs, but most commercial SNP arrays developed for domestic animals are based on random selections of evenly spaced SNPs which may help to lessen this bias^13^. Also, most SNPs on commercial arrays are identified so researchers can screen markers depending on their objectives.

In our study we tested the domestic chicken 600K SNP array on five North American species of *Centrocercus* (sage-grouse) and *Tympanuchus* (sharp-tailed grouse and prairie-chickens) grouse to determine if it was suitable for genetic studies in these taxa. The five species, herein collectively referred to as prairie grouse, are estimated to have diverged from chickens about 37 mya^14^, while the two genera are thought to have split approximately 4.7–9.9 mya^15,16^. The domestic chicken SNP array is based on the *Gallus gallus* 4.0 reference genome and contains 580 954 SNPs, including 21 534 SNPs from coding regions. The SNPs on the array are evenly spaced (mean of 1 748 bases between markers) based on genetic map distance to equalize the density (per cM) of SNPs on micro- and macro-chromosomes^10^. Our cross-species application of chicken SNPs took advantage of the expected high synteny between the genomes of chicken and grouse (see Methods for details) and, to the best of our knowledge, this is the first study to test the Affymetrix 600K SNP array on a species other than *Gallus gallus*.

## Results

**Figure 1:**
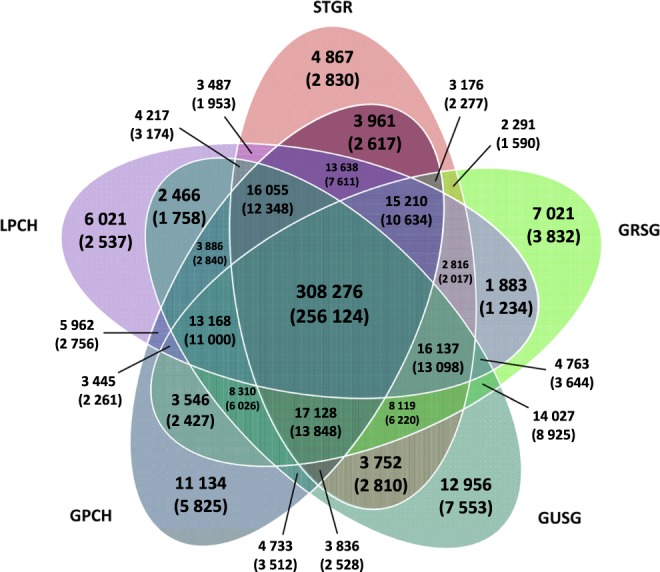
The number of called and polymorphic (given in the parentheses) loci shared among five grouse species. GPCH – Greater Prairie-Chicken, LPCH – Lesser Prairie-Chicken, STRG – Sharp-tailed Grouse, GUSG – Gunnison Sage-Grouse, GRSG – Greater Sage-Grouse.

We successfully genotyped 91% (530 287 of 580 961) of all chicken SNPs on the array (including sex chromosomes), and 53% (308 276 of 530 287) of genotyped SNPs were found in at least one of two sampled individuals of all five species (Fig. 1). Between 73% and 76% of all SNPs on the chip were genotyped per species and 51–52% of SNPs were genotyped for both individuals per species (Table 1). Mean call rate of all genotyped SNPs was 69 ± 0.04%, and 41% of all genotyped SNPs (218 524 of 530 287) were scored in at least nine of ten samples. A total of 488 288 SNPs were genotyped in at least two species (Fig. 1), and of these only 0.2% (1192) showed fixed differences between species (here we only considered a difference as ‘fixed’ if both genotyped individuals of a species were homozygous for an allele that differed from the allele(s) genotyped in the another species). Genotyping success rate of all 21 534 chicken coding SNPs was 63–68% per species.

**Table 1:** Genotyping success of 580 961 SNPs on the 600K Aftymetrix Axiom chicken array and observed heterozygosity of SNPs (H_O_) in five grouse species (two individuals per species).

Species	N genotyped SNPs		N polymorphic SNPs		H_O_
	One individual	Both individuals	One individual	Both individuals	
Greater Prairie-Chicken	435464	298920	289781	212082	0.646
Lesser Prairie-Chicken	421430	294517	276889	207331	0.635
Sharp-tailed Grouse	426966	300764	291893	220205	0.659
Gunnison Sage-Grouse	441829	294531	314318	226139	0.683
Greater Sage-Grouse	429316	294145	294590	217086	0.663
Mean	431001	296575	293494	216569	0.657

Among all genotyped SNPs, 77% (407 798 of 530 287) were polymorphic (had two alleles in the sample). Note that this includes SNPs with estimates from one (53% polymorphic) to ten (85%) samples. On average, 7.2 of the ten samples were genotyped across all polymorphic SNPs. Within each species, 48–54% of all SNPs were polymorphic, and 72–75% of them were genotyped for both individuals per species (Table 1). Average observed heterozygosity was 0.66 (Table 1).

For known autosomal SNPs (N = 546 120), the mean genotyping success rate was 74% averaged across species, and it was similar between all chromosome categories, ranging from 73% for intermediate-chromosomes to 75% for micro-chromosomes (all species combined). The proportion of SNPs among all genotyped loci was similar for all chromosome categories, ranging from 69% for macro-chromosomes to 71% for micro-chromosomes. By contrast, the spacing between adjacent markers differed significantly between autosomal chromosome categories (F_2,133_ = 159.6, P < 0.001). The largest inter-marker distances were found on macro-chromosomes, while the smallest inter-marker distances were found on micro-chromosomes, consistent with the equalization of marker densities across chromosomes (Table 2). In total, 24–26% of markers were located within 1 Kb of adjacent markers and 95–97% of markers were located within 10 Kb of adjacent markers (Figure S1 in the Electronic Supplementary Material, ESM).

**Table 2:** Number of SNPs and mean (±SD) distance between adjacent marker pairs for three groups of autosomal chromosomes in five grouse species. Chromosome groups are based on the chicken genome.

Species	Macro-chromosomes (1–5)		Intermediate-chromosomes (6–10)		Micro-chromosomes (11–28)	
	No. SNPs	Intermarker distance (kbp)	No. SNPs	Intermarker distance (kbp)	No. SNPs	Intermarker distance (kbp)
Greater Prairie-Chicken	154882	3.91 ± 0.32	50064	2.85 ± 0.54	79867	1.98 ± 0.53
Lesser Prairie-Chicken	142156	4.25 ± 0.33	45905	3.10 ± 0.60	74370	2.12 ± 0.59
Sharp-tailed Grouse	149674	4.04 ± 0.31	48574	2.94 ± 0.57	78796	2.00 ± 0.57
Gunnison Sage-Grouse	161105	3.75 ± 0.30	52638	2.71 ± 0.51	83867	1.88 ± 0.52
Greater Sage-Grouse	156312	3.87 ± 0.30	51089	2.79 ± 0.53	81824	1.93 ± 0.54
Mean	152826	3.96 ± 0.31	49654	2.88 ± 0.55	79745	1.98 ± 0.55

**Figure 2:**
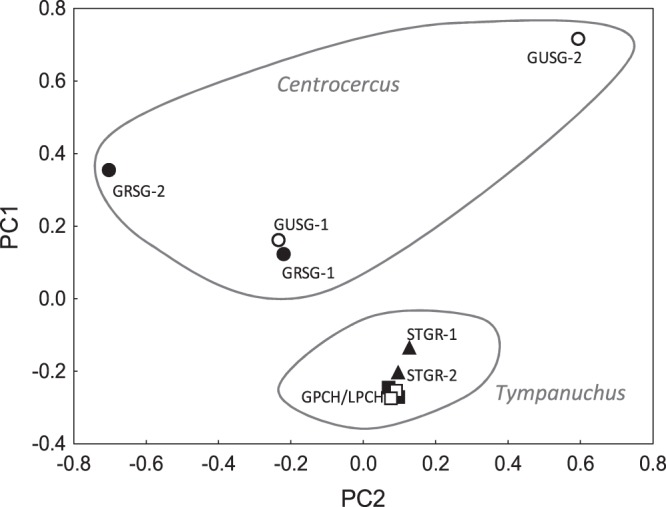
Clustering of five grouse species (two individuals per species) with principal component analysis using autosomal loci. GPCH – Greater Prairie-Chicken (open squares), LPCH – Lesser Prairie-Chicken (filled squares), STRG – Sharp-tailed Grouse (filled triangles), GUSG – Gunnison Sage-Grouse (open circles), GRSG – Greater Sage-Grouse (filled circles).

**Figure 3:**
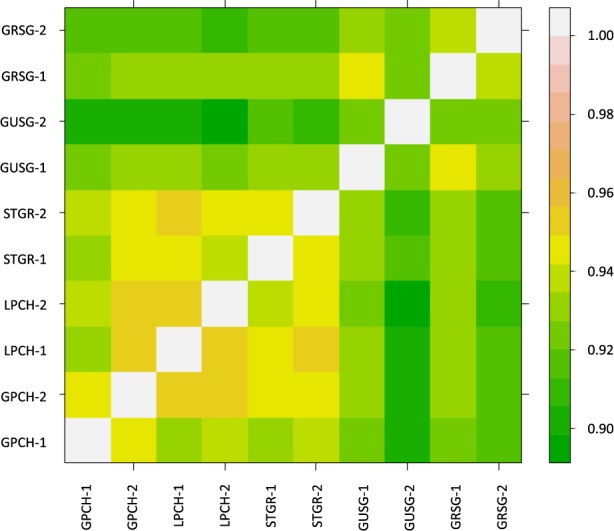
Heatmap of genetic similarity of autosomal loci between individuals of five grouse species. Yellow/orange squares indicate high allele sharing between individuals, green squares indicate low allele sharing. GPCH – Greater Prairie-Chicken, LPCH – Lesser Prairie-Chicken, STGR – Sharp-tailed Grouse, GUSG – Gunnison Sage-Grouse, GRSG – Greater Sage-Grouse.

We conducted a principal component analysis (PCA) to examine variation between species using 386 011 autosomal loci that were polymorphic across our samples. The first PC axis accounted for 21.5% of the variability in the SNP dataset and it distinguished between *Centrocercus* and *Tympanuchus* genera (Fig. 2). However, species within genera were not distinguished with any of the PC axes. On average, there was little separation between genera and species in terms of shared SNPs. The two genera shared 92.0 ± 0.2% of SNPs, and the species within each genus shared 93.2 ± 0.6% (*Centrocercus*) and 94.3 ± 0.2% (*Tympanuchus*) of SNPs (Fig. 3). Similar SNP sharing rates (93.0 ± 0.6% for *Centrocercus* and 94.8% for *Tympanuchus*) were recorded between individuals within species (Fig. 3).

**Figure 4:**
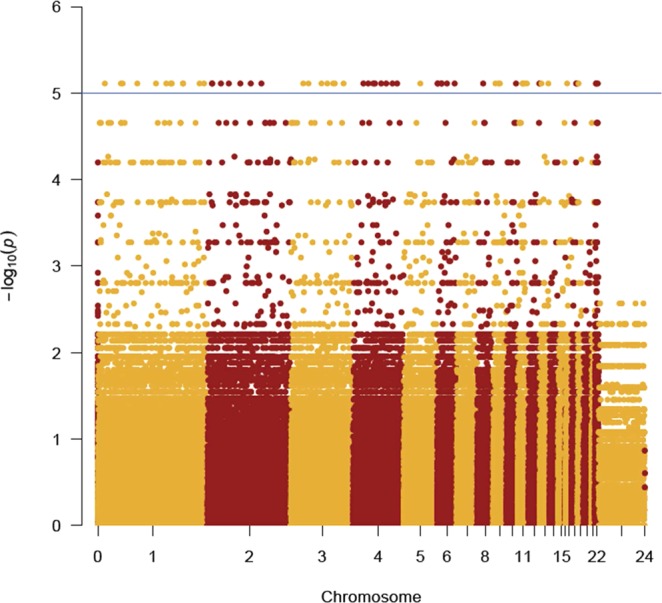
Manhattan plot of differences between *Centrocercus* and *Tympanuchus* grouse at autosomal loci. P values are unadjusted values from the association test in PLINK 1.9. Chromosome are delineated by altering colours. Chromosome numbers are from the Axiom Genotyping Array^10^ include unreferenced locations (0). There were 204 SNPs with P < 1 × 10^−5^ (blue reference line), although the Bonferroni critical level would be 1.26 × 10^−7^ for 350 837 tests.

**Figure 5:**
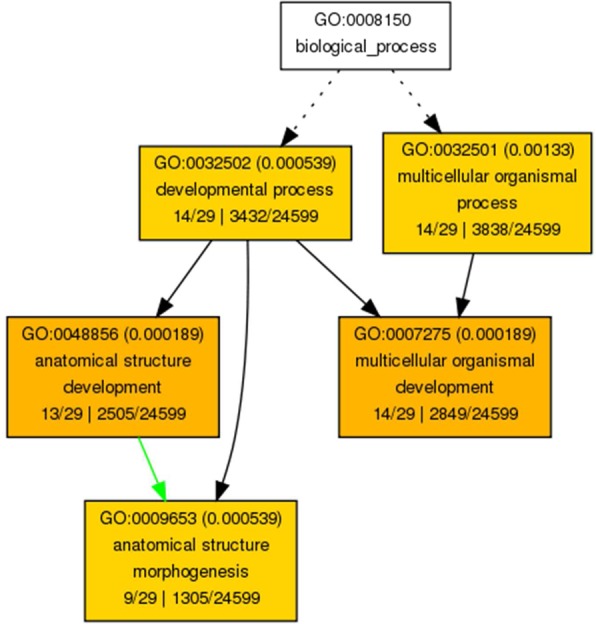
Significant differences between sage and prairie-grouse in terms of enrichment of biological processes. Results are based on 53 SNPs with data from all 10 samples and P = 7.74 × 10^−6^ from the PLINK analysis of both groups. Adjusted P values (FDR < 0.05) from the Fisher’s exact test in the Singular Enrichment analysis (SEA) in AgriGo are indicated in parentheses in the top row within each box. Also in the box are the GO term identifiers, the GO Description (e.g., developmental process), and the frequencies used in the SEA analysis (bottom row). These are the number of items (UNIPROT ids) in the query list mapping to the given GO term (e.g., 14 of the items mapped to developmental process) out of the total in the query list (29 in this case) and number in the background list (i.e., 3432) relative to the total in the reference (i.e., 24599; Axiom array). Solid and dashed lines represent one and two enriched terms at both ends connected by the arrows, respectively.

We also explored potential genetic differences between *Centrocercus* and *Tympanuchus* using the two genera as groups in the association test in PLINK (Fig. 4). Here we tested 350 835 polymorphic SNPs with a minor allele frequency >0.05. As a consequence of the large number of tests we performed (350 835), the Bonferroni critical level (1.26 × 10^−7^) was below the minimum possible *P* value with our sample size (7.74 × 10^−6^) and, consequently, none of the tests was significant after Bonferroni correction. Thus, we used the *P* values as a relative index of importance in separating the two genera. First, we examined the distribution of 5 807 nominally significant (P < 0.05 unadjusted) SNPs that differed between genera. These SNPs were generally distributed evenly across chromosomes relative to the total number of SNPs on each chromosome (median = 0.9% of SNPs were ‘significant’ on each chromosome; range 0.7–1.2%). The only exceptions were small (chr 16; 0.34%) and micro (LGE22C19W2; 1.9%) chromosomes, which is likely an artefact of small numbers of SNPs (<585) on these chromosomes. Next we examined the genes encompassing the most significant SNPs (i.e., P = 7.74 × 10^−6^) that were found in all 10 samples. In this sample of 53 SNPs, the maximum significance level was achieved because all of the individuals in one genus were homozygous for a different allele than individuals in the other genus (Table S1 in ESM). Several of these SNPs, such as Ephrin type-A receptor 7 (EPHA7) and Keratin, type I cytoskeletal 14 (KRT14) are notable because their associated gene (or a closely related one) has been implicated in feather development. To examine the possible function of these 53 SNPs, we used AgriGo for gene ontology analysis and found significant enrichment (FDR < 0.05) in 15 GO terms (Fig. 5). In terms of biological processes, there were strong differences in anatomical structure development (GO: 0048856) and morphogenesis (GO:0009653), as well as various developmental processes (Fig. 5).

## Discussion

Despite the relatively high divergence time between the domestic chicken and grouse (~37 mya^14^), the Affymetrix 600K SNP array produced genotypes for 91% of SNPs in at least one of the five species of grouse and 53% of SNPs across all species of grouse. Even with the loss of about half of the SNPs, the 600K SNP array has potential to produce more SNPs than typical RAD-seq methods (that usually generate <50 000 SNPs), particularly at known coding regions. For example, over 10 K coding SNPs were genotyped in each grouse species, which may encourage studies investigating functional divergence among species or populations. In fact, our cross-application of chicken SNP array in grouse allowed us to identify a set of candidate genes that may be responsible for large phenotypic differences between *Centrocercus* and *Tympanuchus*. Although the number of SNPs we genotyped here was fewer than might be gained through whole genome resequencing (millions), the loci on the array have already been characterized, and therefore less effort is required to filter and identify loci. Our results do suggest, however, that the loci included in the Affymetrix array may not be particularly suitable to infer phylogenetic relationships in grouse, as only 0.2% of all genotyped SNPs showed fixed differences between the species. While the PCA analysis conducted with autosomal loci could separate *Centrocercus* from *Tympanuchus*, it could not distinguish species within genera.

The application of SNP arrays in research on non-model vertebrates has recently become an alternative tool in addition to whole-genome sequencing and RAD-sequencing methods. Although most SNP arrays were originally developed for model or domestic organisms, those same arrays can potentially be applied to wild relatives to obtain large panels of SNP markers^12^. Cross-species application of SNP arrays has been particularly common in mammals^3,11,13,17^, but this approach has not received as much attention in avian research. As far as we are aware, our study provides the first attempt to apply a commercially available SNP array to non-passerine bird species. So far, non-commercial Illumina SNP arrays have been developed for several passerines, such as great tit *Parus major*^5^, house sparrow *Passer domesticus*^6^, and *Ficedula* flycatchers^7^. These SNP arrays allow researchers to genotype tens of thousands (10–50 K) of SNPs, but they may not be effectively applicable in non-passerine birds due to the long evolutionary times since the divergence of these lineages. Thus, genome-wide SNP genotyping in non-passerines has often been based on RAD-seq methods^18–21^. However, RAD-seq applications in non-model bird species do not typically exceed 100 K SNPs^22–24^, and it has been shown that RAD-seq-based genome scans are likely to miss a considerable proportion of loci under selection, as the median density of markers is ca. 4 SNPs/Mb^25^. Thus, the usefulness of RAD-seq data in studies of local adaptation has been recently debated^25^. This problem can potentially be avoided with the Affymetrix SNP array, which has a density of 400–1300 SNPs/Mb at each autosome^10^. In our cross-species study of prairie grouse the Affymetrix array still yielded a density of 250–500 SNPs/Mb, depending on the size of the autosome. Over 21 K annotated SNPs from the Affymetrix array have been identified in exonic regions, while 190 K SNPs have been identified in intronic regions^10^. Thus, considering an even distribution of SNPs across the genome, it can be expected that the majority of the estimated 20 000–23 000 chicken genes^26^ will be covered with the array. The array also includes an additional 266 K SNPs that have been annotated from intergenic regions of the chicken genome^10^. Thus, cross-application of the Affymetrix array in wild avian species may provide researchers with easier means of studying population genetics, local adaptation and genome-wide selection. It is important to note, however, that our small-scale study was designed primarily to test the feasibility of the array, and these other applications may require using greater number of samples.

Our application of the Affymetrix 600K SNP array in prairie grouse yielded surprisingly high levels of polymorphism (48–54% within species, 77% across all five species). This is in stark contrast to previous research on mammals, which showed an exponential decline of shared polymorphism with the time to the last common ancestor^11^. In general, mammalian taxa, which diverged >5 mya shared less than 10% of polymorphic SNPs^11^. Unfortunately, we are aware of no previous attempts to apply SNP arrays across phylogenetically distant avian species, but our analyses suggest that some bird lineages may be much more conservative in the retention of shared polymorphism than mammals. Although this hypothesis certainly needs more empirical testing, it appears supported by the high similarity of draft genomic sequences of several grouse species to the reference chicken genome^27–29^. This high similarity between chicken and grouse also suggests that mispriming was not a likely bias in using the SNP array. At the same time, we acknowledge that genome synteny is unlikely to provide an exclusive explanation for high levels of shared polymorphism between grouse and chicken. In mammals, very high genome synteny between closely related taxa may not necessarily be associated with high levels of shared polymorphism. For example, cross-species application of SNPs developed for the domestic sheep *Ovis aries* in a recently diverged (ca. 2 mya) wild species, the bighorn sheep *Ovis canadensis*, showed very low level of retained polymorphism (less than 2%), despite high genome synteny between the two species^17^.

We also acknowledge that the application of the chicken SNP array in wild grouse could carry some risk of ascertainment bias, where the cross-amplified panel of SNPs is not representative of the overall pattern of genetic diversity in the study species. This could be caused by over-representation of evolutionarily old shared polymorphisms and under-representation of rare and novel variants^11^. Also, application of arrays originally developed for domestic animals in wildlife could introduce an over-representation of SNPs resulting from artificial selection. While these limitations have to be carefully considered while using SNP arrays, it should also be acknowledged that the problem of ascertainment bias is not restricted to SNP arrays, but is inherent in any marker development and can be compensated for during data analysis^11,30^. Cross-amplified loci should always be subject to the process of initial filtering, which may include removal of loci that show low allele frequencies (usually with MAF <5%), deviate from neutrality, or show strong linkage disequilibrium (LD) within a given basepair window. The latter approach (LD based SNP pruning) is particularly useful for reducing the effects of ascertainment bias, especially when estimating differentiation measures between populations (e.g. genetic distances), inbreeding coefficients or kinships^31^. The low sample size in this study prevented us from exploring these possibilities in any detail, but we recommend using these filtering steps in future studies that apply SNP arrays across species based on larger sample sizes.

Successful application of the Affymetrix 600K SNP array in North American prairie grouse suggests that the same method could be used for genotyping other galliform species. Chickens (Tribe Gallini) and grouse (Tribe Tetraonini) are thought to have diverged relatively early in the radiation of the family Phasianidae. Thus, it seems likely that the Affymetrix array might be successfully applied across the entire Phasianidae clade. However, galliform species that are more distantly related to chickens, such as New World quail (Odontophoridae) or guineafowl (Numididae) would probably yield lower genotyping success than in the case of grouse. Cross-application of the array in phasianids may provide an effective tool for molecular studies of this group, including those focused on conservation related issues. In general, phasianids are one of the most threatened groups of birds, since they are often affected by direct exploitation, as well as challenged with severe habitat loss and degradation^32^. Nearly 40% of all 187 extant phasianid species face some level of conservation risk and 12 species have been classified by the International Union for the Conservation of Nature (IUCN) as endangered (EN) or critically endangered (CR), and thus facing a very high risk of extinction in the near or immediate future^33^. All five taxa studied here are of conservation concern; Gunnison sage-grouse is classified as endangered and both species of prairie-chicken are classified as vulnerable by IUCN. As a consequence of habitat loss, these species have undergone long-term and rapid population declines, and have increasingly small and fragmented ranges^34^. All five of the study species are under active conservation programs, including translocations of birds to restore genetic variation^35^, or captive-breeding and reintroduction programs^19,36^. Thus, we suggest that the Affymetrix 600K SNP array may become a new and efficient tool to obtain genome-wide data that are increasingly important for the management of threatened populations of grouse and other phasianids^35,37,38^.

Our study provided support for high levels of shared polymorphisms between grouse species, especially within genera. This pattern seems to be consistent with previous phylogenetic studies on prairie grouse that suggest a recent divergence among species within the genera *Tympanuchus* and *Centrocercus*. For example, the fossil-calibrated species tree using both autosomal and Z-linked nuclear loci estimated the time of divergence, within *Tympanuchus*, between the greater prairie-chicken and lesser prairie-chicken at less than 300 K years^39^. Also, autosomal and mtDNA loci showed extensive allele sharing among *Tympanuchus* species with minimal haplotype clustering corresponding with taxonomy^31^. A low degree of reciprocal monophyly between species within *Centrocercus* and *Tympanuchus* genera was also found based on the analysis of several innate immune genes^40^. This pattern was attributed to incomplete lineage sorting and female-biased introgression, as hybridization between *Tympanuchus* species has been widely reported in the areas of geographic overlap^41–43^ and female hybrids have been documented breeding in the wild^39^. Although our results are in general agreement with previous research, we explicitly acknowledge that the statistical power of our analyses was low due to small sample sizes (two individuals per species, ten individuals in total). Consequently, analyses using larger sample sizes are needed to investigate whether the Affymetrix 600k SNP array could be used to investigate phylogenetic relationships within *Centrocercus* and *Tympanuchus*. Despite these limitations, our study suggests that even with hundreds of thousands of autosomal SNPs, there may be relatively few SNPs contributing to fixed differences between prairie grouse species, especially within *Tympanuchus*. This view is reinforced by several recent studies that find relatively small regions of the genome are responsible for the large phenotypic differences between some closely related species of birds^44–46^. At the same time, we acknowledge that having *de novo* SNPs discovered directly in prairie grouse could change this pattern and reveal more fixed difference between our study species.

The sex chromosomes were excluded from our clustering analysis because we had an unbalanced mix of both sexes among species in our small sample set that impacted our ability to investigate phylogenetic relationships (samples clustered largely based on sex; Figure S2 in ESM). As sex chromosomes often show a higher divergence than autosomes between species^47^, they may contribute disproportionately to maintaining species boundaries, as previously reported for prairie grouse^39,48^. Since the chicken SNP array includes over 25 K Z-linked loci, future studies with larger sample sizes and a more balanced representation of sexes among taxa could use the array to investigate phylogenetic relationships among grouse (and other galliform taxa).

We identified differences between sage-grouse (*Centrocercus*) and prairie-chickens (*Tympanuchus*; Fig. 2), which differ most notably in body mass and plumage, even though our study had small sample sizes (four and six individuals in each genus, respectively). Sage-grouse are over two times heavier than prairie-chickens (2.4–3.9 kg versus 0.8–1 kg, respectively). In addition, sage-grouse have much longer tail feathers and exaggerated feathers around the neck (a white ruff) and head (filoplumes), which are used in mating displays^49^ and are not present in prairie-chickens. Recent genome scans of pigeons and doves with feather crests (or exaggerated neck feathers similar to sage-grouse) have found associations between those traits and SNPs at the Ephrin receptor B2 (EphB2) gene, which is a receptor for tyrosine kinase. The Eph family of molecules has 14 members, which play a role in tissue patterning and morphogenesis^50^ and, in birds, influence the development of the feather cytoskeleton^51,52^. In our study Ephrin type-A receptor 7 (EphA7) showed strong differences between the two genera of grouse. The A and B type receptors are generally thought to be activated by different types of ligands, but there appears to be high levels of interaction between them (e.g., between EphB2 and EphA5^53^), suggesting that other Eph receptors could also be involved in feather development. We also found differences between the two genera of grouse at several other genes that have been associated with feather morphology (KRT14, a member of the keratin gene family which is associated with frizzled feathers; KRT75^54^), melanin pigmentation (FZD4 and LIMK1^55^) and body mass (miR-16^56^) in birds. GO analysis revealed that these genes were primarily enriched in processes that influenced morphology and development, consistent with the large phenotypic differences between these two genera of grouse.

Although the results of our clustering and gene ontology analyses must be treated with caution because of low sample sizes, the main conclusion that emerges from our study is that the Affymetrix 600K array is a promising tool for future research on molecular ecology and conservation genetics of grouse. SNP arrays have the potential to advance studies of closely related non-model species, but currently they may be too expensive for the budgets of many researchers (e.g., $205 per sample at GeenSeek, Lincoln, NE, USA). Over time, however, these costs may decrease. One of the advantages of the array is that it enables much easier access to an annotated genome and the traits associated with variable SNPs. Such information is becoming critically important for population genetic studies of animals in the wild and endangered species, in particular. Our results should provide some encouragement for other researchers willing to investigate the utility of using commercial SNP arrays in other non-model species.

## Methods

We collected samples from all five species of North American prairie grouse in the genera *Centrocercus* (greater sage-grouse, *C. urophasianus*, and Gunnison sage-grouse, *C. minimus*) and *Tympanuchus* (greater prairie-chicken, *T. cupido*; lesser prairie-chicken, *T. pallidicinctus*; and sharp-tailed grouse, *T. phasianellus*). Blood samples were taken for DNA extraction from two individuals per species. Samples were collected from wild populations between 1996 and 2006 in Colorado (greater sage-grouse and Gunnison sage-grouse), Kansas (lesser prairie-chicken), Minnesota (greater-prairie chicken), and North Dakota (sharp-tailed grouse). The sampling of sexes was unbalanced among species, resulting in a total of six females (two greater sage-grouse, two greater prairie-chicken, one lesser prairie-chicken, and one sharp-tailed grouse) and four males (two Gunnison sage-grouse, one lesser prairie-chicken, and one sharp-tailed grouse). Genomic DNA was extracted using protocols described elsewhere^39,57,58^. Sample collection complied with the current laws of the USA and with the approval of IACUC committees at Colorado Department of Parks and Wildlife, Kansas State University, and the Univ. of Wisconsin-Milwaukee.

All ten individuals from the five species were genotyped using the Axiom Genome-Wide Chicken Genotyping array (Affymetrix, Santa Clara, CA, USA, now owned by ThermoFisher Scientific) that was originally developed for the domestic chicken^10^. The Affymetrix 600K array was originally developed to contain a large catalogue of SNPs that are segregating within diverse populations of the domestic chicken and to address a variety of purposes, including the detection of genetic associations with complex traits, fine mapping of quantitative trait loci, detecting signatures of selection, exploitation of the linkage disequilibrium structure of the genome, and implementation of genomic selection^10^. The array contains exactly 580 961 biallelic SNPs across 28 autosomes, two sex chromosomes, and two linkage groups (LGE64 and LGE22C19W28_E50C23) that have not yet been assigned to a chromosome, but are expected to reside on micro-chromosomes^59^. Genotyping was performed by GeneSeek (Lincoln, NE, USA) using Genotyping Console Software v. 4.2 (Affymetrix). A minimum quality control score >0.82 (default), which is based on the signal to noise ratio, was used to filter each sample. The ten grouse samples were run on a single Axiom array (96 samples total) that also included domestic chicken samples. The grouse samples were analysed separately from the chicken samples following Affymetrix guidelines, because otherwise the statistical thresholds used to call SNPs would be based on the much larger chicken sample and this would lead to the exclusion of many SNPs among the grouse samples.

Although we have no direct information on the genome organization in our study grouse species, birds in general are notable for possessing high level of karyotypic conservation and synteny across chromosomes^60,61^. Traditionally, genomic analyses with phasianids often take advantage of their close relationship with the domestic chicken because it is well characterized in terms of genomic organization. To date, draft genomes of various grouse species^28,29^, including sage-grouse^27^, have been assembled and mapped to the reference chicken genome. For example, the draft genome of black grouse *Tetrao tetrix* had high synteny with the main chicken autosomal chromosomes 1–28, as well as sex chromosomes^28^. No major genomic rearrangements have been reported for grouse, when compared to the chicken genome^27,28^. Thus, based on the previous research, we assumed that genomes of our study grouse species shared high synteny with the chicken genome.

We performed a principal component analysis (PCA) of the SNP genotypes to examine patterns of genetic diversity in the 10 samples. This analysis used SNPs that were successfully genotyped in at least one individual (see Results). PCA was conducted using the *snpgdsPCA* function in the *SNPRelate* package^62^ developed for the R statistical environment^63^. The *gdsfmt* package^62^ in R was used for managing the genomic data structure (GDS) files. To avoid confounding sex and species differences in our small sample, we removed the sex chromosomes (Z and W) from the analyses comparing different taxa (n = 21 787 SNPs removed), but we included a separate analysis of the sex chromosomes in the Electronic Supplementary Material. Previous studies have indicated that the sex chromosomes are likely important in speciation and tend to differentiate faster than autosomes between species^47,64^. Indeed, a previous study of *Centrocercus* showed that the Gunnison and greater sage-grouse showed much stronger differentiation at SNPs on the Z chromosome than on the autosomes^48^ (see also Galla & Johnson^39^ for comparisons between *Tympanuchus* spp.). Allele sharing rates were calculated as average identity-by-state (IBS) pairwise identities using the *snpgdsIBS* function in *SNPRelate*.

Following the PCA, we used PLINK v. 1.9^65^ to test individual SNPs for an association with taxonomy, here specifically between genera because of the PCA results (see Results). To understand the biological function of SNPs that differed between genera, we performed gene ontology (GO) analysis as implemented in Agrigo (http://bioinfo.cau.edu.cn/agriGO/index.php^66^) using the chicken Affymetrix array data for the genetic background. Fisher’s exact test with a false discovery rate (FDR) <0.05 was used to test for significant enrichment in each GO term. All values are presented as means ± SE.

## Supplementary information

Electronic Supplementary Material

## Data Availability

The data used in this study are available from the corresponding authors upon request.

## References

[1] Kardos M, Husby A, McFarlane SE, Qvarnström A, Ellegren H (2015). Whole genome resequencing of extreme phenotypes in collared flycatchers highlights the difficulty of detecting quantitative trait loci in natural populations. Mol. Ecol. Res..

[2] Pertoldi C (2010). Genome variability in European and American bison detected using the BovineSNP50 BeadChip. Conserv. Genet..

[3] Haynes GD, Latch EK (2012). Identification of novel single nucleotide polymorphisms (SNPs) in deer (*Odocoileus* spp.) using the BovineSNP50 BeadChip. PLOS ONE.

[4] Kharzinova VR (2015). A study of applicability of SNP chips developed for bovine and ovine species to whole-genome analysis of reindeer *Rangifer tarandus*. J. Hered..

[5] van Bers NEM (2012). The design and cross-population application of a genome-wide SNP chip for the great tit *Parus major*. Mol. Ecol. Res..

[6] Hagen IJ (2013). The easy road to genome-wide medium density SNP screening in a non-model species: development and application of a 10 K SNP-chip for the house sparrow (*Passer domesticus*). Mol. Ecol. Res..

[7] Kawakami T (2014). Estimation of linkage disequilibrium and interspecific gene flow in *Ficedula* flycatchers by a newly developed 50 k single-nucleotide polymorphism array. Mol. Ecol. Res..

[8] Jetz W, Thomas GH, Joy JB, Hartmann K, Mooers AO (2012). The global diversity of birds in space and time. Nature.

[9] Claramunt S, Cracraft J (2015). A new time tree reveals Earth history’s imprint on the evolution of modern birds. Sci. Adv..

[10] Kranis A (2013). Development of a high density 600K SNP genotyping array for chicken. BMC Genomics.

[11] Miller JM, Kijas JW, Heaton MP, McEwan JC, Coltman DW (2012). Consistent divergence times and allele sharing measured from cross-species application of SNP chips developed for three domestic species. Mol. Ecol. Res..

[12] McCue ME (2012). A high density SNP array for the domestic horse and extant Perissodactyla: utility for association mapping, genetic diversity, and phylogeny studies. PLOS Genet..

[13] Hoffman JI, Thorne MAS, McEwing R, Forcada J, Ogden R (2013). Cross-amplification and validation of SNPs conserved over 44 million years between seals and dogs. PLOS ONE.

[14] Kumar S, Stecher G, Suleski M, Hedges SB (2017). TimeTree: A resource for timelines, timetrees, and divergence times. Mol. Biol. Evol..

[15] Stein RW, Brown JW, Mooers AØ (2015). A molecular genetic time scale demonstrates Cretaceous origins and multiple diversification rate shifts within the order Galliformes (Aves). Mol. Phylogenet. Evol..

[16] Persons NW, Hosner PA, Meiklejohn KA, Braun EL, Kimball RT (2016). Sorting out relationships among the grouse and ptarmigan using intron, mitochondrial, and ultra-conserved element sequences. Mol. Phylogenet. Evol..

[17] Miller JM, Poissant J, Kijas JW, Coltman DW (2011). A genome‐wide set of SNPs detects population substructure and long range linkage disequilibrium in wild sheep. Mol. Ecol. Res..

[18] Dierickx EG, Shultz AJ, Sato F, Hiraoka T, Edwards SV (2015). Morphological and genomic comparisons of Hawaiian and Japanese Black‐footed Albatrosses (*Phoebastria nigripes*) using double digest RADseq: Implications for conservation. Evol. Appl..

[19] Bateson ZW (2016). Specific alleles at immune genes, rather than genome‐wide heterozygosity, are related to immunity and survival in the critically endangered Attwater’s prairie‐chicken. Mol. Ecol..

[20] Cristofari R (2016). Full circumpolar migration ensures evolutionary unity in the Emperor penguin. Nat. Commun..

[21] Tigano A, Shultz AJ, Edwards SV, Robertson GJ, Friesen VL (2017). Outlier analyses to test for local adaptation to breeding grounds in a migratory arctic seabird. Ecol. Evol..

[22] Bourgeois YX (2013). Mass production of SNP markers in a nonmodel passerine bird through RAD sequencing and contig mapping to the zebra finch genome. Mol. Ecol. Res..

[23] Shultz AJ, Baker AJ, Hill GE, Nolan PM, Edwards SV (2016). SNPs across time and space: population genomic signatures of founder events and epizootics in the House Finch (*Haemorhous mexicanus*). Ecol. Evol..

[24] Szulkin M, Gagnaire PA, Bierne N, Charmantier A (2016). Population genomic footprints of fine‐scale differentiation between habitats in Mediterranean blue tits. Mol. Ecol..

[25] Lowry DB (2017). Breaking RAD: an evaluation of the utility of restriction site‐associated DNA sequencing for genome scans of adaptation. Mol. Ecol. Res..

[26] International Chicken Genome Sequencing Consortium (2004). Sequence and comparative analysis of the chicken genome provide unique perspectives on vertebrate evolution. Nature.

[27] Card DC (2014). Two low coverage bird genomes and a comparison of reference-guided versus de novo genome assemblies. PLoS ONE.

[28] Wang B, Ekblom R, Bunikis I, Siitari H, Höglund J (2014). Whole genome sequencing of the black grouse (*Tetrao tetrix*): reference guided assembly suggests faster-Z and MHC evolution. BMC Genomics.

[29] Kozma R, Melsted P, Magnússon KP, Höglund J (2016). Looking into the past–the reaction of three grouse species to climate change over the last million years using whole genome sequences. Mol. Ecol..

[30] Clark AG, Hubisz MJ, Bustamante CD, Williamson SH, Nielsen R (2005). Ascertainment bias in studies of human genome-wide polymorphism. Genome Res..

[31] Malomane DK (2018). Efficiency of different strategies to mitigate ascertainment bias when using SNP panels in diversity studies. BMC Genomics.

[32] Keane A, Brook M, de L, McGowan PJK (2005). Correlates of extinction risk and hunting pressure in gamebirds (Galliformes). Biol. Conserv..

[33] Winkler, D. W., Billerman, S. M. & Lovette, I. J. Bird Families of the World: An Invitation to the Spectacular Diversity of Birds. *Lynx Edicions, Barcelona* (2015).

[34] BirdLife International. IUCN Red List for birds Downloaded from http://www.birdlife.org (2019).

[35] Bateson ZW (2014). Genetic restoration of a threatened population of greater prairie-chickens. Biol. Conserv..

[36] Apa AD, Wiechman LA (2015). Captive‐rearing of Gunnison sage‐grouse from egg collection to adulthood to foster proactive conservation and recovery of a conservation‐reliant species. Zoo Biol..

[37] Garson PJ, Young L, Kaul R (1992). Ecology and conservation of the cheer pheasant *Catreus wallichii*: studies in the wild and the progress of a reintroduction project. Biol. Conserv..

[38] Sokos CK, Birtsas PK, Tsachalidis EP (2008). The aims of galliforms release and choice of techniques. Wildl. Biol..

[39] Galla SJ, Johnson JA (2015). Differential introgression and effective size of marker type influence phylogenetic inference of a recently divergent avian group (Phasianidae: *Tympanuchus*. Mol. Phylogenet. Evol..

[40] Minias P (2018). Extensive shared polymorphism at non-MHC immune genes in recently diverged North American prairie grouse. Immunogenetics.

[41] Bain MR, Farley GH (2002). Apparent hybrid prairie-chickens in a zone of geographic overlap. Condor.

[42] Augustine JK, Trauba DR (2015). Potential for behavioral reproductive isolation between greater prairie-chickens and sharp-tailed grouse in west-central Minnesota. J. Ethol..

[43] Oyler-McCance SJ (2016). Rangewide genetic analysis of lesser prairie-chicken reveals population structure, range expansion, and possible introgression. Conserv. Genet..

[44] Lamichhaney S (2016). A beak size locus in Darwin’s finches facilitated character displacement during a drought. Science.

[45] Toews DP (2016). Plumage genes and little else distinguish the genomes of hybridizing warblers. Curr. Biol..

[46] Campagna L (2017). Repeated divergent selection on pigmentation genes in a rapid finch radiation. Sci. Adv..

[47] Irwin Darren E. (2018). Sex chromosomes and speciation in birds and other ZW systems. Molecular Ecology.

[48] Oyler-McCance SJ, Cornman RS, Jones KL, Fike JA (2015). Z chromosome divergence, polymorphism and relative effective population size in a genus of lekking birds. Heredity.

[49] Johnsgard, P. A. The grouse of the world. (University of Nebraska Press, Lincoln, 1983).

[50] Park JE, Son AI, Zhou R (2013). Roles of EphA2 in development and disease. Genes.

[51] Shapiro MD (2013). Genomic diversity and evolution of the head crest in the rock pigeon. Science.

[52] Vickrey AI, Domyan ET, Horvath MP, Shapiro MD (2015). Convergent evolution of head crests in two domesticated columbids is associated with different missense mutations in EphB2. Mol. Biol. Evol..

[53] Himanen J-P (2004). Repelling class discrimination: ephrin-A5 binds to and activates EphB2 receptor signaling. Nat. Neurosci..

[54] Ng CS (2012). The chicken frizzle feather is due to an α-Keratin (KRT75) mutation that causes a defective rachis. PLOS Genet..

[55] Pauli M (2015). De novo assembly of the dual transcriptomes of a polymorphic raptor species and its malarial parasite. BMC Genomics.

[56] Jia X, Lin H, Nie Q, Zhang X, Lamont SJ (2016). A short insertion mutation disrupts genesis of miR-16 and causes increased body weight in domesticated chicken. Sci. Rep..

[57] Bellinger MR, Johnson JA, Toepfer J, Dunn PO (2003). Loss of genetic variation in greater prairie chickens following a population bottleneck in Wisconsin, USA. Conserv. Biol..

[58] Oyler-McCance SJ, St John J, Taylor SE, Apa AD, Quinn TW (2005). Population genetics of Gunnison Sage-Grouse: Implications for management. J. Wildl. Manage..

[59] Guizard S, Piégu B, Arensburger P, Guillou F, Bigot Y (2016). Deep landscape update of dispersed and tandem repeats in the genome model of the red jungle fowl, *Gallus gallus*, using a series of de novo investigating tools. BMC Genomics.

[60] Shetty S, Griffin DK, Graves JAM (1999). Comparative painting reveals strong chromosome homology over 80 million years of bird evolution. Chromosome Res..

[61] Ellegren H (2010). Evolutionary stasis: the stable chromosomes of birds. Trends Ecol. Evol..

[62] Zheng X (2012). A high-performance computing toolset for relatedness and principal component analysis of SNP data. Bioinformatics.

[63] R Development Core Team R: A language and environment for statistical computing (R Foundation for Statistical Computing, Vienna, Austria, 2013).

[64] Charlesworth B, Coyne JA, Barton NH (1987). The relative rates of evolution of sex chromosomes and autosomes. Am. Nat..

[65] Purcell S (2007). PLINK: A tool set for whole-genome association and population-based linkage analyses. Am. J. Hum. Genet..

[66] Du Z, Zhou X, Ling Y, Zhang Z, Su Z (2010). AgriGO: a GO analysis toolkit for the agricultural community. Nucleic Acids Res..

