# Supplementary material for: Evaluation of a Chicken 600K SNP genotyping array in non-model species of grouse
Source: Sci Rep. 2019 Apr 23;9:6407. doi: 10.1038/s41598-019-42885-5 (PMC6478925; doi:10.1038/s41598-019-42885-5)

Electronic Supplementary Material

Evaluation of a Chicken 600K SNP genotyping array in non-model species of grouse

Piotr Minias[1], Peter O. Dunn[1,2], Linda A. Whittingham[2], Jeff A. Johnson[3], Sara J. Oyler-McCance[4]

[1]Department of Biodiversity Studies and Bioeducation, Faculty of Biology and Environmental Protection, University of Łódź, Banacha 1/3, 90-237 Łódź, Poland.[2]Behavioral and Molecular Ecology Group, Department of Biological Sciences, University of Wisconsin-Milwaukee, Milwaukee, Wisconsin, USA. [3]Department of Biological Sciences, Institute of Applied Sciences, University of North Texas, Denton, Texas, USA. [4]Fort Collins Science Center, US Geological Survey, Ft. Collins, Colorado, USA. Correspondence and requests for materials should be addressed to P.M. (email: pminias@op.pl)

Table S1. Significant SNPs from PLINK analysis of *Centrocercus* and *Tympanuchus* grouse. Only the top 53 SNPs with P= 7.74x10-6 and data from all 10 samples are listed. Probe set ID is from the Axiom genotyping array. UniProt ID is the identifier used in Agrigo. Gene and protein names are from UniProtKB (www.uniprot.org).

Chromosome	Chromosomal Position	Probe Set ID	UniProt ID	Gene name	Protein name
1	12339820	AX-76119323	E1BQU3	TMEM60	Transmembrane protein 60
1	40984019	AX-76814552	Q08856	MYF5	Myogenic factor 5
1	44088043	AX-76696920	P34743	BTG1	Protein BTG1 (B-cell translocation gene 1 protein)
1	70157809	AX-76188790	E1C874	ARHGAP8	Uncharacterized protein; regul. of GTPase activity
1	121727709	AX-76153770	E1BTB6	FANCB	Fanconi anemia complementation group B
1	129552681	AX-76910711	Q5ZHN4	RP2	Protein XRP2
1	131561744	AX-75256307	A0A1D5PV73	TMEM131	Transmembrane protein 131
1	151226552	AX-76548580	F1NZH0	SLITRK1	SLIT and NTRK like family member 1
1	151990504	AX-76526534	F1NZG9	SPRY2	Protein sprouty homolog 2
1	181095815	AX-75647528	Q90729	GluR4	GluR4 flip (Fragment)
1	188952325	AX-75860279	Q9IA05	FZD4	Frizzled-4
2	7941559	AX-76736576	Q05917	EN2	Homeobox protein engrailed-2
2	8327792	AX-75674558	Q7ZUA6	LMBR1	Limb region 1 protein homolog
2	23818951	AX-76679165	P02467	COL1A2	collagen, type I, alpha 2
2	37852023	AX-76719746	P68306	THRB	Thyroid hormone receptor beta
2	95882959	AX-75705893	Q90763	CDH7	Cadherin-7
3	21644457	AX-76436129	F1NHX4	RCOR3	REST corepressor 3
3	30982939	AX-77186071	Q5ZJD7	YIPF4	Protein YIPF4
3	54132527	AX-76166696	B4XN22	IFNGR1	Interferon gamma receptor 1
3	64498467	AX-75246732	A0A1D5NW46	HDAC2	Histone deacetylase (EC 3.5.1.98)
3	74824206	AX-76618838	O42422	EPHA7	Ephrin type-A receptor 7
3	77922074	AX-75508086	A0A1D5PDD1	FAM46A	Family with sequence similarity 46 member A
3	93517926	AX-75453192	A0A1D5P9I6	COLEC11	Collectin-11
4	18313145	AX-76160200	A0A1L1RU31	IDS	Uncharacterized protein; iduronate-2-sulfatase activity
4	26956046	AX-75752996	Q90ZT4	PCDH10	OL-protocadherin isoform
4	35992948	AX-75910958	R9PXN7	HPGDS	Hematopoietic prostaglandin D synthase
4	38982075	AX-76658216	O93417	CASP3	Caspase-3
4	49922162	AX-76963319	Q4AE91	NPFFR	Neuropeptide FF receptor
4	58881859	AX-75593281	Q7T2Z5	UNC5C	Netrin receptor UNC5C
4	69999103	AX-76897144	Q5F3A4	RELL1	RELT-like protein 1
4	78327809	AX-75364100	A0A1L1RPP3	STX18	Syntaxin 18
5	29459826	AX-75195517	A0A1D5PDP0	RYR3	Ryanodine receptor 3
6	607292	AX-75251789	A0A1D5PRA4	CCSER2	Coiled-coil serine rich protein 2
6	7911561	AX-77169919	Q5ZJ69	ARID5B	AT-rich interactive domain-containing protein 5B
6	17361196	AX-76555415	F1NZV4	FAM21A	Uncharacterized protein
6	31077540	AX-76946050	Q5ZHW3	BUB3	BUB3, mitotic checkpoint protein
8	10705158	AX-76968100	Q5MNV6	SLC30A7	Zinc transporter 7
9	3712485	AX-76083981	D0UYB8	CRYGS	Crystallin gamma S
9	9341483	AX-76059086	C7E4R0	---not avail.	NYGGF4-like protein
9	11750524	AX-75676115	Q8JJC0	ZIC1	Zinc finger protein ZIC 1
10	17044848	AX-75744191	Q9DD46	ALDH6	Aldehyde dehydrogenase
11	12172380	AX-76652635	O93319	CDH11	Cadherin-11
11	16530707	AX-76967718	Q5ILG9	HNF4beta	Hepatic nuclear factor 4beta
12	19302869	AX-77058229	Q5ZIP0	RCJMB04_24j13	Uncharacterized protein
13	3113359	AX-76635112	O73888	HPGDS	Hematopoietic prostaglandin D synthase
13	15395835	AX-76588300	F6RS52	C13H5ORF15	Chromosome 5 open reading frame 15
17	2769302	AX-75235495	A0A1D5PJ88	TNC	Tenascin
18	8353485	AX-77129364	Q5ZM91	PRKAR1A	cAMP-dependent protein kinase type I-alpha regulatory subunit
18	10260966	AX-75446956	A0A1D5NY12	SPAG9	Sperm associated antigen 9
19	1488001	AX-75635156	Q8QFP8	LIMK1	LIM domain kinase 1
25	1534800	AX-75297408	A0A1D5PV83	RPRD2	Uncharacterized protein
27	1704795	AX-76504125	F1NU79	PSMC5	Proteasome 26S subunit, ATPase 5
27	4603231	AX-75298886	A0A1D5PZ89	KRT14	Keratin, type I cytoskeletal 14

Figure S1. Frequency distribution of distance between adjacent marker pairs. Across-species means ± 1.96*SE presented.

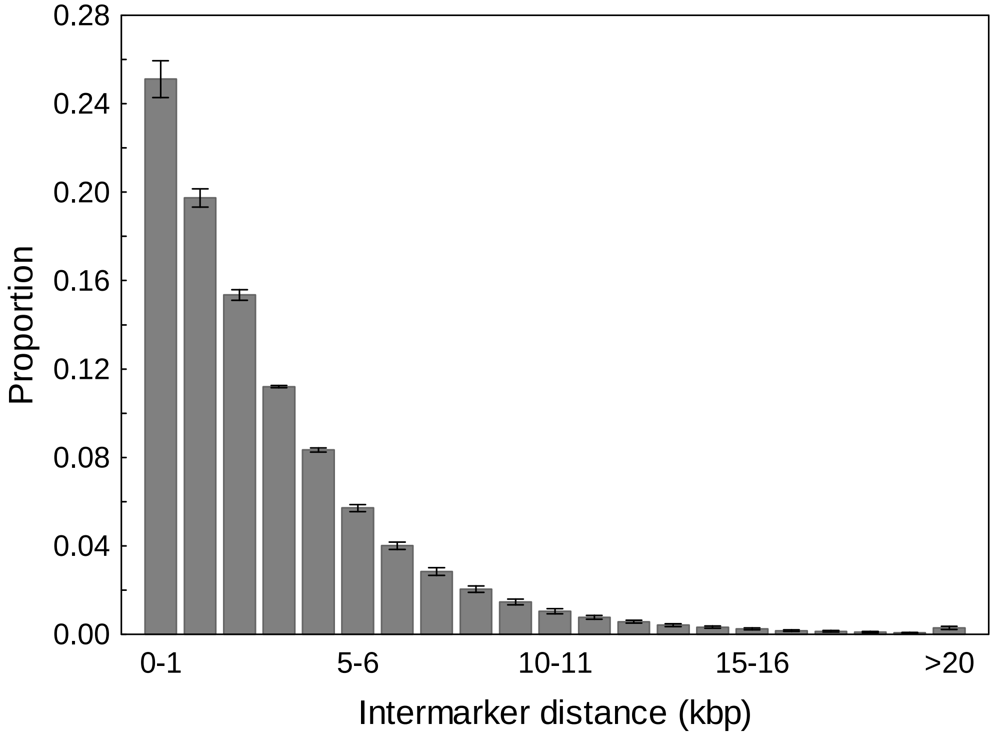

Figure S2. Clustering of five grouse species (two individuals per species) with principal component analysis using sex chromosome loci. GPCH – Greater Prairie-Chicken, LPCH – Lesser Prairie-Chicken, STRG – Sharp-tailed Grouse, GUSG – Gunnison Sage-Grouse, GRSG – Greater Sage-Grouse.

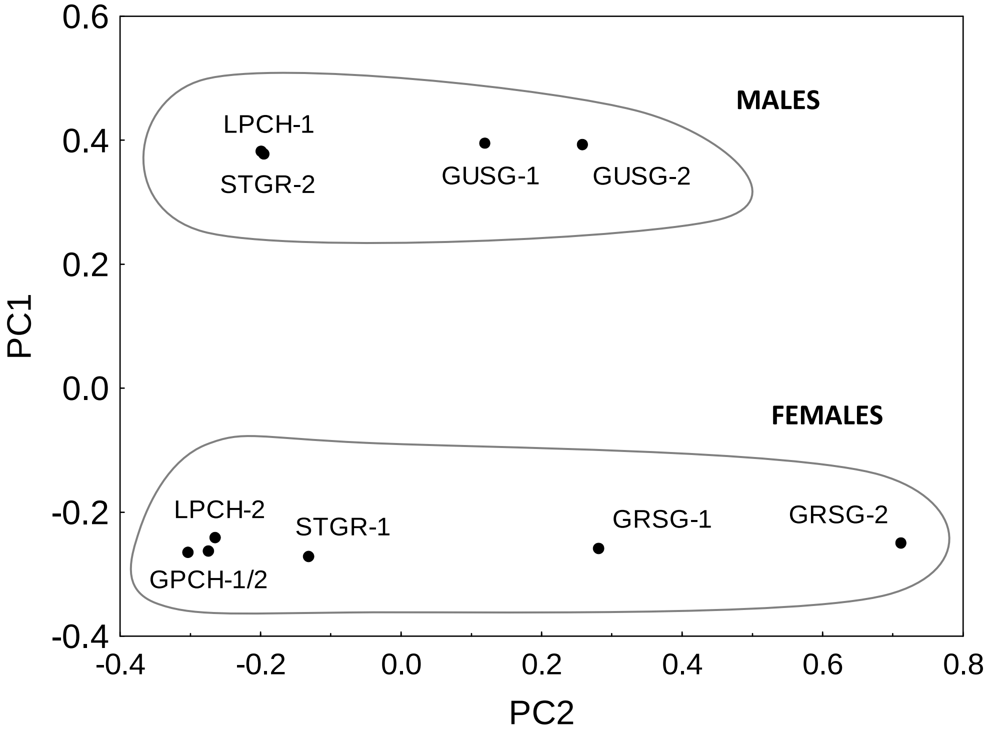

Supplement: Supplementary file 1 — Electronic Supplementary Material [file 41598_2019_42885_MOESM1_ESM.pdf]
